# Prevalence and variability of current depressive disorder in 27 European countries: a population-based study

**DOI:** 10.1016/S2468-2667(21)00047-5

**Published:** 2021-05-04

**Authors:** Jorge Arias-de la Torre, Gemma Vilagut, Amy Ronaldson, Antoni Serrano-Blanco, Vicente Martín, Michele Peters, Jose M Valderas, Alex Dregan, Jordi Alonso

**Affiliations:** a Department of Psychological Medicine, Division of Academic Psychiatry, Institute of Psychiatry, Psychology & Neuroscience, King's College London, London, UK; b CIBER Epidemiology and Public Health, Madrid, Spain; c Health Services Research Group, Hospital del Mar Medical Research Institute, Barcelona, Spain; d Institut de Recerca Sant Joan de Déu, Parc Sanitari Sant Joan de Déu, Barcelona, Spain; e Institute of Biomedicine (IBIOMED), Universidad de León, León, Spain; f Nuffield Department of Population Health, University of Oxford, Oxford, UK; g Health Services and Policy Research Group, Exeter Collaboration for Academic Primary Care (APEx), NIHR School for Primary Care Research, NIHR Applied Research Collaboration South West Peninsula (PenARC), University of Exeter, Exeter, UK; h Department of Experimental and Health Sciences, Pompeu Fabra University, Barcelona, Spain

## Abstract

**Background:**

We aimed to estimate the prevalence of current depressive disorder in 27 European countries, and to explore differences in prevalence between European countries and by gender.

**Methods:**

In this population-based study, we analysed data from respondents living in 27 European countries who were included in the second wave of the European Health Interview Survey, collected between 2013 and 2015. We assessed the prevalence of current depressive disorder using the eight-item Patient Health Questionnaire (PHQ-8), with depressive disorder defined as a PHQ-8 score of 10 or higher. Prevalence estimates and 95% CIs were calculated for all 27 countries overall and for each country individually. We assessed variation in prevalence (country *vs* the rest of Europe) using crude and adjusted prevalence ratios obtained from negative binomial regression models. We did all analyses for the total sample and stratified by gender.

**Findings:**

Our analysis sample comprised 258 888 individuals, of whom 117 310 (weighted proportion 47·8%) were men and 141 578 (52·2%) were women. The overall prevalence of current depressive disorder was 6·38% (95% CI 6·24–6·52) with important variation across countries, ranging from 2·58% (2·14–3·02) in the Czech Republic to 10·33% (9·33–11·32) in Iceland. Prevalence was higher in women (7·74% [7·53–7·95]) than in men (4·89% [4·71–5·08]), with clear gender differences for all countries except Finland and Croatia. Compared with the other European countries in our sample, those with the highest adjusted prevalence ratios were Germany (1·80 [1·71–1·89]) and Luxembourg (1·50 [1·35–1·66]), and those with the lowest adjusted prevalence ratios were Slovakia (0·28 [0·24–0·33]) and the Czech Republic (0·32 [0·27–0·38]).

**Interpretation:**

Depressive disorders, although common across Europe, vary substantially in prevalence between countries. These results could be a baseline for monitoring the prevalence of current depressive disorder both at a country level in Europe and for planning health-care resources and services.

**Funding:**

UK Medical Research Council and CIBER Epidemiology and Public Health (CIBERESP).

## Introduction

Depressive disorders are a major cause of disability, increasing the risk of premature mortality, decreasing quality of life, and creating a substantial burden on health systems.^1^, ^2^, ^3^ Different estimates show that these disorders could affect more than 300 million people worldwide.^4^, ^5^, ^6^, ^7^ However, these estimates have high temporal and geographical variation, making a periodical assessment for each country or region necessary.

Multiple studies have been done in Europe with a focus on the assessment of the prevalence of depression.^5^, ^8^, ^9^ These studies suggested that the prevalence of depression across Europe is between 5% and 10%, with potentially large differences between countries (eg, 10% in the UK and 7% in Germany).^5^, ^9^ Furthermore, differences in prevalence over time within the same countries might exist.^5^, ^10^ Despite the use of large and, in some cases, representative samples in these studies, their results are difficult to compare due to differences in the variables collected and the assessed populations. Although some studies have assessed multiple European countries, most of them do not cover the whole of Europe.^5^, ^9^, ^10^ Additionally, the use of diagnostic codes in some estimations could not pick up the full extent of the prevalence of depression, missing some cases, such as those with little access to health care.^4^, ^5^, ^9^ An updated assessment of the prevalence of depressive disorders, including a basic set of relevant covariates, covering most European countries, and using a valid, reliable, and well established instrument for the assessment of depression, is essential for monitoring and planning health resources and services, both at a European-wide and a country-specific level.^11^

Different demographic and socioeconomic factors, such as self-identified gender, age, and social deprivation, have been linked to the prevalence of depression.^10^, ^12^, ^13^, ^14^ These factors vary widely within and across countries and could contribute to international variation in the prevalence of depression. In Europe, socioeconomic differences between countries are pronounced. Additionally, geographical and sociopolitical factors might explain differences between countries in the prevalence of depression.^15^ An in-depth analysis of the current prevalence of depression from an inter-country (European-wide) and intra-country (countrywide) perspective, taking into account these factors, would provide crucial data that could be used to inform health policy for the prevention of depression at the European level, and might allow proposal of specific preventive measures adapted to the particularities of those countries where prevalence is higher relative to other countries.

**Research in context**

**Evidence before this study**

We searched PubMed without language restrictions for observational studies published from database inception until July 27, 2020, reporting prevalence estimates for any European country using population-wide or representative samples. We used the MeSH terms “depress*” AND “prevalence” AND “Europe” AND (“representative*” OR “population*”). Multiple studies have been done in European countries examining the prevalence of depression. Evidence from specific countries suggests differences between them and over time. Additionally, some studies suggested that these differences could be affected by variability between countries in sociodemographic, health-related, and lifestyle factors. Despite the use of large, population-wide, and, in some cases, representative samples in these studies, their results were difficult to compare due to differences in the measures used to assess depression, the type of variables considered, and the populations studied.

**Added value of this study**

To our knowledge, this is the first study to assess differences in the prevalence of current depressive disorder in Europe using primary representative data of the general population in the community at country level. Using data for 27 European countries collected between 2013 and 2015 as part of the second wave of the European Health Interview Survey, we found that the overall prevalence of current depressive disorder is high (6·38%), with important variation across European countries. The highest prevalence estimate for an individual country was approximately four times higher than the lowest estimate and the highest prevalences were seen in countries with high economic development. Additionally, we found that prevalence of depression was higher among women than among men, with a clear difference in prevalence according to gender in all countries except for Finland and Croatia.

**Implications of all the available evidence**

Our findings could be a reference and baseline for the monitoring of the prevalence of current depressive disorder, the planning of health-care resources and services, and the development of screening and other preventive strategies both at a European and country level. Moreover, our analyses might be considered a replicable approach to assessing and comparing prevalence of depression between European countries.

We aimed to estimate the prevalence of current depressive disorder in 27 European countries and assess differences between the prevalence for each specific country and that of the whole European sample.

## Methods

### Study design and population

In this population-level cross-sectional study, we used data from the second wave of the European Health Interview Survey (EHIS-2). The EHIS-2 is a European-wide survey, representative at country level, which includes all 27 EU member states plus the UK, Norway, Iceland, and Turkey.^16^, ^17^ The target population of the surveys comprised individuals aged 15 years and older (16 years and older for Sweden and the UK) living in private households residing in the territory of the country at the time of the data collection. Depending on the country, the EHIS-2 sample was selected using different sampling methods (including single-stage and multiple-stage strategies) and data were collected from 2013 to 2015 through different procedures (postal, face-to-face, telephone, internet, or mixed methods). To maximise the response rate and minimise possible bias and differences in responses related to the method of data collection, interviews were done by trained interviewers. The non-response rate by country in EHIS-2, before substitutions (ie, use of reserve sample participants when main sample participants were non-contactable), ranged from 16% to 70%, and did not exceed 40% in 17 of 27 countries.^16^ However, compensation methods for non-response rates were used to reach the estimated minimum effective sample size in those countries where this was not achieved.^16^ The overall ratio between the actual effective sample size and the estimated minimum effective sample size in the countries included in this study ranged from 0·81 (Greece) to 2·17 (Latvia).^16^ Additionally, the mixed sampling methods were accounted for in our analyses through weighting.^16^ More detailed information about the specific sampling methods, response rates, and how EHIS-2 determined the sample to achieve comparability and representativeness can be found in the quality report and methodological manual of EHIS-2.^16^, ^17^

EHIS-2 anonymised microdata were provided by Eurostat after we signed an agreement about security, confidentiality, accessibility, and use of data. Ethical approval was not required for this research due to the public and anonymous nature of the data we used.

### Data collection and analysis

The EHIS-2 questionnaire collects data on health status, socioeconomic status, mental health (including the eight-item Patient Health Questionnaire [PHQ-8]), and other determinants of health (eg, gender and age). Conceptual equivalence of all items across countries was ensured through appropriate modifications where needed (eg, wording modifications to adapt questions to the specificities of the national languages).

We assessed the prevalence of current depressive disorder using PHQ-8, an instrument based on Diagnostic and Statistical Manual of Mental Disorders (DSM) IV criteria, which has been shown to be valid and reliable for assessing current depressive disorder in the general population.^18^, ^19^, ^20^, ^21^ Because of the absence of availability of PHQ-8 variables in the microdata files from Belgium, the Netherlands, and Spain,^16^ data from these countries were not included in this study. Additionally, because of the paucity of information about their quality,^16^ data from Turkey were also excluded. Hence, only 27 of 31 countries included in the EHIS-2 had available data on current depressive disorder and so were included in our analyses.

The PHQ-8 is a self-reported questionnaire composed of eight items, which correspond to the DSM-IV diagnostic criteria for major depressive episode excluding thoughts of death and suicide. The recall period corresponds to the previous 2 weeks and the response scale ranged from 0 (not at all) to 3 (nearly every day). The PHQ-8 score is calculated by adding the answers for each of the items and ranges from 0 to 24. Based on results from previous validation studies,^19^, ^21^ for this study we considered a PHQ-8 score of 10 or higher to be positive for current depressive disorder (sensitivity over 85% and specificity over 88%).^19^, ^21^ Furthermore, the prevalence of depression was assessed using the scoring algorithm of PHQ-8 for major depressive syndrome and other depressive syndrome and a self-reported item (self-reported depression [SRD]; a single question on experiencing depression in the past 12 months: “During the past 12 months, have you had depression? Yes/No”) were calculated as sensitivity analyses.^17^, ^18^ Only interviews with complete information on depression using the PHQ-8 were included. Additionally, due to their known association with depression and usual inclusion in populational health studies, the following covariates were selected for the analyses: sociodemographic factors (gender, age, birthplace, degree of urbanisation of the individuals' residence area, net monthly income of the household equivalised for each country, and educational level), health-related factors (long-standing illness and general activity limitation), and lifestyle factors (body-mass index [BMI], intake of fruit and vegetables, smoking status, and physical activity [measured as number of days per week doing activities that cause at least a small increase in breathing or heart rate for at least 10 min continuously]).

### Statistical analysis

We calculated the prevalence and 95% CI for current depressive disorder for each specific country and across all the included countries. Additionally, in a supplementary analysis, we calculated the distribution by country of the scores for each PHQ-8 item (dichotomous: 0 = item score of <2; 1 = item score of ≥2). To assess differences in the prevalence of current depressive disorder between each specific country and the rest of the included countries, we calculated the crude prevalence ratio from a bivariable negative binomial regression model and we calculated the adjusted prevalence ratio from a multivariable negative binomial regression model. To assess differences in prevalence, we adjusted the negative binomial model each country using a dummy variable (country *vs* the rest of Europe as reference) as the main explanatory factor. Because of their known association with depression, we included all covariates in the multivariable models using a theory-driven approach (ie, an approach based on previous evidence and clinical relevance). We assessed the absence of multicollinearity by calculating the variance inflation factor for each of the multivariable models. We used the Wald test to assess the statistical significance of the variable country within the models. We did all analyses for the whole population and stratified by gender. In all our analyses, we took into account the final individual weights derived from the sampling strategy for each country (using the Taylor linearisation method for survey data to be as conservative as possible in the estimations). Moreover, because the population analysed was calculated by summing the populations of each country and expanded using individual weights, the use of final individual weights allowed us to compare estimates between each country and the rest of the included countries. The proportion of missing data for the variables in the study population ranged from 0% to 5·3% for household income from an open question and multivariable normality could not be assumed. To deal with missing data, we did multiple imputation using chained equations with five imputations.^22^ Finally, we did sensitivity analyses calculating the prevalence and prevalence ratios (country *vs* all other included countries) using the algorithm scoring of PHQ-8, the SRD indicator, and geographically pooled data.

We did all analyses using Stata version 16.1.

### Role of the funding source

The funder of the study had no role in study design, data collection, data analysis, data interpretation, or writing of the report.

## Results

316 333 individuals were in the EHIS-2 dataset, of whom 39 608 were excluded because they were not in the countries included in our analysis and 17 837 were excluded because they did not meet the inclusion criteria. Our final analysis sample comprised 258 888 respondents to the EHIS-2 (117 310 [weighted proportion 47·8%] men and 141 578 [52·2%] women) who completed the PHQ-8 (table 1). The prevalence of current depressive disorder for our European cohort was 6·38% (95% CI 6·24–6·52) overall, 4·89% (4·71–5·08) for men, and 7·74% (7·53–7·95) for women (table 2). The subgroups with the highest prevalence of current depressive disorder were generally those who were older (aged ≥75 years), born in a non-EU country, living in densely populated areas, had a long-standing illness, were severely limited in their activity, had a BMI of 30 kg/m^2^ or more or 18·5 kg/m^2^ or less, were daily smokers, and were sedentary (table 2). Additionally, the prevalence of depression decreased as income and educational level increased and as fruit and vegetable consumption decreased in the total population. These patterns were consistent in both genders (table 2).

**Table 1:** General characteristics of the study population overall and by gender

		**Total population (n=258 888)**	**Men (n=117 310)**	**Women (n=141 578)**
Age group, years				
	15–29	19·89% (19·64–20·14)	20·79% (20·42–21·16)	19·06% (18·74–19·39)
	30–44	24·81% (24·57–25·06)	25·35% (24·98–25·72)	24·32% (23·99–24·64)
	45–59	25·79% (25·55–26·02)	26·31% (25·95–26·66)	25·31% (24·99–25·63)
	60–74	19·60% (19·40–19·80)	19·09% (18·80–19·38)	20·06% (19·78–20·34)
	≥75	9·91% (9·75–10·08)	8·46% (8·25–8·68)	11·25% (11·00–11·49)
Country of birth				
	Native born	91·87% (91·70–92·04)	92·26% (92·02–92·50)	91·50% (91·27–91·74)
	Another EU member state	3·15% (3·05–3·26)	2·85% (2·70–3·00)	3·43% (3·28–3·58)
	Non-EU country	4·98% (4·84–5·12)	4·89% (4·69–5·09)	5·07% (4·88–5·26)
Residence area population				
	Densely	36·06% (35·79–36·33)	35·20% (34·80–35·60)	36·85% (36·48–37·22)
	Intermediate	33·05% (32·79–33·31)	33·25% (32·86–33·65)	32·86% (32·51–33·22)
	Thinly	30·89% (30·64–31·14)	31·55% (31·18–31·92)	30·28% (29·95–30·62)
Net monthly income (quintiles)				
	1 (lower income)	19·29% (19·07–19·52)	17·57% (17·25–17·88)	20·88% (20·56–21·19)
	2	19·44% (19·21–19·66)	18·13% (17·80–18·46)	20·63% (20·31–20·95)
	3	19·80% (19·57–20·02)	19·46% (19·11–19·80)	20·11% (19·81–20·42)
	4	20·44% (20·21–20·66)	21·49% (21·15–21·83)	19·48% (19·18–19·77)
	5 (higher income)	21·03% (20·80–21·27)	23·36% (23·00–23·72)	18·90% (18·60–19·20)
Long-standing illness		42·61% (42·33–42·88)	39·72% (39·32–40·12)	45·26% (44·88–45·64)
General activity limitation				
	Severely limited	6·57% (6·44–6·71)	6·07% (5·88–6·26)	7·04% (6·84–7·23)
	Limited but not severely	19·10% (18·89–19·32)	17·31% (17·01–17·60)	20·75% (20·45–21·05)
	Not limited	74·32% (74·09–74·56)	76·63% (76·29–76·96)	72·21% (71·88–72·54)
Educational level				
	Primary or lower	9·66% (9·51–9·82)	8·38% (8·17–8·59)	10·84% (10·62–11·06)
	Secondary	64·28% (64·01–64·55)	65·09% (64·69–65·49)	63·54% (63·18–63·90)
	Tertiary	26·05% (25·80–26·31)	26·53% (26·15–26·91)	25·62% (25·29–25·95)
Body-mass index, kg/m^2^				
	<18·5	2·81% (2·71–2·91)	1·46% (1·35–1·57)	4·05% (3·89–4·21)
	18·5–24·9	47·05% (46·77–47·33)	41·22% (40·81–41·63)	52·40% (52·01–52·78)
	25–29·9	34·85% (34·58–35·13)	41·77% (41·36–41·63)	28·51% (28·17–28·85)
	≥30	15·29% (15·09–15·49)	15·55% (15·25–15·85)	15·05% (14·78–15·32)
Diet (fruit and vegetables)				
	Daily	65·13% (64·87–65·40)	59·08% (58·68–59·49)	70·68% (70·34–71·02)
	4–6 times per week	20·36% (20·14–20·59)	22·58% (22·24–22·93)	18·33% (18·04–18·61)
	1–3 times per week	12·15% (11·97–12·33)	15·20% (14·91–15·49)	9·34% (9·13–9·56)
	Less than once per week	2·36% (2·27–2·45)	3·14% (2·99–3·28)	1·65% (1·55–1·74)
Smoking status				
	Daily smoking	18·92% (18·70–19·14)	22·82% (22·48–23·16)	15·33% (15·06–15·61)
	Occasional smoking	5·02% (4·89–5·14)	5·83% (5·63–6·02)	4·27% (4·12–4·42)
	No smoking	76·07% (75·86–76·31)	71·35% (70·98–71·72)	80·40% (80·09–80·70)
Days per week physically active				
	0	55·43% (55·14–55·72)	53·22% (52·80–53·64)	57·46% (57·08–57·85)
	1	9·01% (8·83–9·18)	8·93% (8·67–9·19)	9·08% (8·84–9·32)
	2	11·50% (11·31–11·69)	11·44% (11·16–11·72)	11·55% (11·30–11·81)
	3	9·58% (9·40–9·75)	10·18% (9·91–10·44)	9·03% (8·80–9·25)
	4	4·48% (4·36–4·60)	5·14% (4·95–5·33)	3·87% (3·72–4·02)
	5	3·78% (3·67–3·90)	4·29% (4·11–4·48)	3·31% (3·17–3·45)
	6	1·62% (1·55–1·70)	2·01% (1·88–2·14)	1·27% (1·18–1·35)
	7	4·60% (4·49–4·73)	4·80% (4·62–4·98)	4·43% (4·27–4·59)

Data are weighted proportion with 95% CI in parentheses.

**Table 2:** Prevalence of current depressive disorder by demographic and clinical characteristics, overall and by sex

		**Total population (n=15 757)**	**Men (n=5305)**	**Women (n=10 452)**
Overall		6·38% (6·24–6·52)	4·89% (4·71–5·08)	7·74% (7·53–7·95)
Age group, years				
	15–29	5·26% (4·93–5·59)	3·63% (3·22–4·04)	6·89% (6·37–7·40)
	30–44	4·86% (4·59–5·12)	4·21% (3·83–4·59)	5·48% (5·11–5·84)
	45–59	7·10% (6·82–7·39)	5·69% (5·31–6·07)	8·45% (8·03–8·87)
	60–74	5·85% (5·58–6·12)	4·65% (4·30–4·99)	6·90% (6·50–7·30)
	≥75	11·59% (11·01–12·16)	8·11% (7·38–8·84)	13·99% (13·16–14·81)
Country of birth				
	Native born	6·26% (6·12–6·41)	4·78% (4·59–4·98)	7·63% (7·41–7·84)
	Another EU member state	6·41% (5·55–7·26)	5·07% (3·86–6·29)	7·43% (6·25–8·60)
	Non-EU country	8·53% (7·73–9·32)	6·85% (5·79–7·92)	10·01% (8·83–11·18)
Residence area population				
	Densely	6·95% (6·69–7·21)	5·54% (5·20–5·89)	8·18% (7·81–8·55)
	Intermediate	6·16% (5·92–6·41)	4·73% (4·41–5·06)	7·49% (7·12–7·85)
	Thinly	5·94% (5·72–6·17)	4·33% (4·05–4·62)	7·48% (7·13–7·83)
Net monthly income (quintiles)				
	1 (lower income)	10·62% (10·20–11·04)	9·22% (8·60–9·84)	11·70% (11·13–12·28)
	2	7·68% (7·33–8·03)	5·88% (5·41–6·35)	9·13% (8·62–9·64)
	3	5·99% (5·69–6·30)	4·45% (4·06–4·85)	7·36% (6·90–7·82)
	4	4·67% (4·39–4·95)	3·60% (3·25–3·96)	5·76% (5·34–6·18)
	5 (higher income)	3·31% (3·08–3·53)	2·43% (2·15–2·72)	4·30% (3·92–4·67)
Long-standing illness				
	Yes	11·80% (11·52–12·08)	9·69% (9·29–10·08)	13·50% (13·10–13·89)
	No	2·35% (2·23–2·48)	1·74% (1·58–1·89)	2·98% (2·79–3·17)
General activity limitation				
	Severely limited	34·58% (33·55–35·61)	30·60% (29·09–32·12)	37·72% (36·33–39·12)
	Limited but not severely	10·97% (10·57–11·37)	8·85% (8·28–9·43)	12·59% (12·04–13·14)
	Not limited	2·70% (2·59–2·82)	1·96% (1·81–2·11)	3·42% (3·25–3·60)
Educational level				
	Primary or lower	11·48% (10·93–12·02)	8·07% (7·33–8·80)	13·90% (13·14–14·66)
	Secondary	6·52% (6·34–6·70)	5·11% (4·88–5·35)	7·84% (7·57–8·11)
	Tertiary	4·13% (3·90–4·37)	3·35% (3·03–3·67)	4·88% (4·54–5·21)
Body-mass index, kg/m^2^				
	<18·5	9·09% (8·00–10·17)	7·90% (6·03–9·76)	9·48% (8·17–10·79)
	18·5–24·9	5·49% (5·29–5·69)	4·41% (4·13–4·70)	6·26% (6·00–6·53)
	25–29·9	5·73% (5·51–5·95)	4·21% (3·95–4·47)	7·77% (7·39–8·16)
	≥30	10·10% (9·65–10·55)	7·72% (7·13–8·32)	12·35% (11·68–13·02)
Diet (fruit and vegetables)				
	Daily	5·60% (5·43–5·76)	3·96% (3·74–4·17)	6·85% (6·61–7·09)
	4–6 times per week	6·24% (5·93–6·55)	4·54% (4·15–4·92)	8·17% (7·67–8·67)
	1–3 times per week	9·06% (8·58–9·54)	7·43% (6·85–8·02)	11·48% (10·67–12·29)
	Less than once per week	15·34% (13·96–16·71)	12·79% (11·08–14·51)	19·77% (17·47–22·07)
Smoking status				
	Daily smoking	8·31% (7·93–8·69)	6·30% (5·85–6·75)	11·05% (10·40–11·70)
	Occasional smoking	6·18% (5·54–6·82)	5·22% (4·38–6·05)	7·38% (6·39–8·38)
	No smoking	5·91% (5·76–6·07)	4·42% (4·21–4·62)	7·13% (6·90–7·35)
Days per week physically active				
	0	7·86% (7·66–8·07)	6·27% (5·99–6·55)	9·22% (8·92–9·51)
	1	5·02% (4·57–5·47)	3·59% (3·03–4·16)	6·31% (5·62–7·00)
	2	4·41% (4·04–4·78)	3·03% (2·56–3·50)	5·66% (5·10–6·21)
	3	4·34% (3·92–4·76)	3·51% (2·95–4·06)	5·20% (4·55–5·84)
	4	4·17% (3·55–4·80)	3·22% (2·46–3·98)	5·33% (4·31–6·35)
	5	4·05% (3·48–4·63)	2·61% (1·95–3·27)	5·77% (4·78–6·75)
	6	4·48% (3·43–5·53)	3·18% (1·88–4·47)	6·37% (4·63–8·11)
	7	5·07% (4·48–5·67)	4·02% (3·25–4·79)	6·12% (5·21–7·02)

Data are for all respondents with current depressive disorder, defined as a PHQ-8 score of 10 or higher. Data are weighted percentage with 95% CI in parentheses. EU member states include the 28 members states as of 2015. PHQ-8=eight item Patient Health Questionnaire.

The prevalence of current depressive disorder by country ranged from 2·56% (95% CI 2·16–2·97) in Slovakia to 10·33% (9·33–11·32) in Iceland (table 3). Prevalence was higher among women than among men, both overall and in all countries, with clear gender differences seen in prevalence for all countries except for Finland (4·86% [3·88–5·83] for men and 5·59% [4·70–6·48] for women) and Croatia (3·08% [2·40–3·76] for men and 3·38% [2·71–4·05] for women). Gender differences in the prevalence of current depressive disorder were particularly prominent in Iceland and Portugal, with the prevalence being twice as high in women than in men. A sensitivity analysis using the algorithm scoring for the PHQ-8 and the SRD indicator showed slightly higher but consistent prevalence estimates compared with using a PHQ-8 cutoff score of 10 or higher (appendix p 2) Additionally, we calculated the distribution of scores for each PHQ-8 item by country, and found that, overall, the item with the highest prevalence of positives was item 4 (ie, feeling tired or having little energy; 11·90% [11·72–12·08]) and the item with the lowest prevalence was item 8 (ie, moving or speaking so slowly that other people could have noticed? Or the opposite—being so fidgety or restless that you have been moving around a lot more than usual; 2·56% [2·47–2·65]; appendix p 3).

**Table 3:** Prevalence of current depressive disorder in 27 European countries, overall and by gender

	**Total population (n=258 888)**		**Men (n=117 310)**		**Women (n=141 578)**	
	n	Prevalence (95% CI)	n	Prevalence (95% CI)	n	Prevalence (95% CI)
Austria	15 701	4·29% (3·82–4·77)	6953	3·41% (2·71–4·12)	8748	5·13% (4·49–5·78)
Bulgaria	5258	6·53% (5·88–7·19)	2458	4·90% (4·07–5·73)	2800	7·99% (7·00–8·98)
Croatia	5016	3·24% (2·76–3·72)	2350	3·08% (2·40–3·76)	2666	3·38% (2·71–4·05)
Cyprus	4695	3·31% (2·80–3·83)	2216	2·48% (1·84–3·13)	2479	4·06% (3·27–4·85)
Czech Republic	6607	2·58% (2·14–3·02)	2776	1·71% (1·17–2·25)	3831	3·39% (2·71–4·07)
Denmark	5449	7·17% (6·45–7·89)	2489	4·88% (3·99–5·78)	2960	9·43% (8·31–10·54)
Estonia	5439	6·64% (5·95–7·34)	2310	4·73% (3·81–5·65)	3129	8·26% (7·25–9·28)
Finland	5146	5·23% (4·58–5·89)	2210	4·86% (3·88–5·83)	2936	5·59% (4·70–6·48)
France	14 191	7·03% (6·54–7·51)	6843	4·78% (4·20–5·35)	7348	9·12% (8·35–9·89)
Germany	24 404	9·24% (8·82–9·66)	11 084	7·69% (7·12–8·27)	13 320	10·73% (10·13–11·34)
Greece	7834	3·39% (2·92–3·85)	3216	2·57% (1·90–3·23)	4618	4·13% (3·48–4·77)
Hungary	5777	7·98% (7·26–8·69)	2678	5·89% (4·97–6·81)	3099	9·82% (8·76–10·88)
Iceland	3812	10·33% (9·33–11·32)	1872	6·67% (5·48–7·86)	1940	14·02% (12·43–15·61)
Ireland	9046	7·67% (6·89–8·44)	4078	6·50% (5·45–7·54)	4968	8·80% (7·66–9·93)
Italy	21 934	3·81% (3·55–4·07)	10 358	2·62% (2·30–2·94)	11 576	4·88% (4·48–5·29)
Latvia	6607	4·61% (4·08–5·13)	2692	2·97% (2·31–3·63)	3915	5·90% (5·11–6·68)
Lithuania	4982	3·01% (2·56–3·46)	1956	1·75% (1·20–2·30)	3026	4·05% (3·37–4·73)
Luxembourg	3629	9·74% (8·76–10·72)	1657	8·12% (6·77–9·46)	1972	11·34% (9·91–12·76)
Malta	3974	3·27% (2·69–3·84)	1899	2·16% (1·49–2·84)	2075	4·38% (3·45–5·30)
Norway	8069	5·21% (4·62–5·79)	4059	4·01% (3·29–4·73)	4010	6·41% (5·49–7·33)
Poland	22 076	4·31% (4·01–4·62)	9620	3·18% (2·79–3·57)	12 456	5·29% (4·83–5·74)
Portugal	17 974	9·15% (8·54–9·77)	7850	4·54% (3·91–5·18)	10 124	13·24% (12·24–14·23)
Romania	16 422	4·38% (4·04–4·72)	7768	3·82% (3·36–4·27)	8654	4·91% (4·41–5·40)
Slovakia	5489	2·56% (2·16–2·97)	2454	1·84% (1·32–2·36)	3035	3·24% (2·62–3·85)
Slovenia	5914	5·50% (4·89–6·12)	2653	3·84% (3·05–4·63)	3261	7·12% (6·17–8·06)
Sweden	5737	8·75% (7·98–9·51)	3024	6·46% (5·56–7·36)	2713	11·08% (9·85–12·31)
UK	17 706	7·40% (6·90–7·89)	7787	6·08% (5·41–6·76)	9919	8·53% (7·81–9·25)

Data are number of respondents without weighting and weighted prevalence with 95% CI in parentheses.

In all countries, the crude prevalence ratio of current depressive disorder varied significantly when compared with the rest of the European population, except for Bulgaria and Estonia (figure). In adjusted analyses, Bulgaria, Norway, and Romania were the only countries that did not significantly differ from the European average (figure). After adjustment for covariates, all countries with significantly high crude prevalence ratios remained significantly increased, whereas the adjusted prevalence ratio for Estonia was significantly reduced compared with the European average (appendix p 4). The countries with the highest adjusted prevalence ratios compared with the rest of Europe were Germany (1·80 [1·71–1·89]) and Luxembourg (1·50 [1·35–1·66]) and the countries with the lowest adjusted prevalence ratios were Slovakia (0·28 [0·24–0·33]) and the Czech Republic (0·32 [0·27–0·38]; appendix p 4). In sensitivity analyses, the crude and adjusted prevalence ratio estimates for each country calculated using the algorithm scoring method for the PHQ-8 and the SRD indicator were consistent with those obtained using the PHQ-8 cutoff score of 10 or higher in most of countries and, particularly, in countries with the highest and lowest adjusted prevalence ratios (appendix p 5). Additionally, sensitivity analyses using geographically pooled data showed that the geographical distribution of countries might have influenced the prevalences estimated in our main analyses, with countries in western Europe having a significantly higher prevalence of current depressive disorder than countries in the other regions of Europe (appendix p 6). Among men, the highest prevalence ratios compared with the rest of Europe were in Germany and Ireland and the lowest prevalence ratios were in the Czech Republic and Slovakia (table 4). Among women, the highest prevalence ratios compared with the rest of Europe were in Germany and Luxembourg and the lowest prevalence ratios were in Slovakia and the Czech Republic (table 4).

**Figure:**
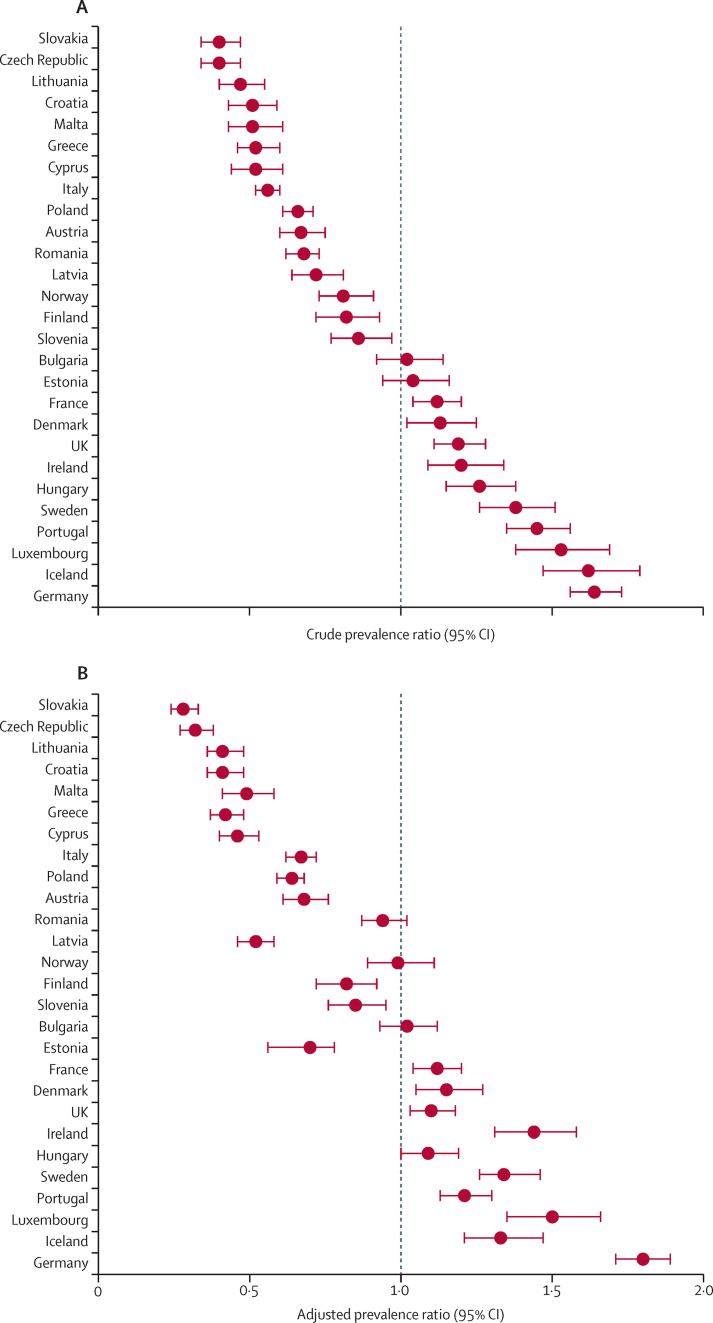
Crude prevalence ratios (A) and adjusted prevalence ratios (B) for current depressive disorder (country *vs* the rest of Europe) Datapoints are prevalence ratio with whiskers showing 95% CIs. Adjusted prevalence ratio is adjusted for gender, age, country of birth, residence area, net monthly income of the household (equivalised for the different countries), long-standing illness, general activity limitation educational level, body-mass index, diet (fruits and vegetables consumption), smoking status, and days per week being physically active. Crude adjusted prevalence ratios are in the appendix (p 4).

**Table 4:** Prevalence ratio of current depressive disorder (country *vs* the rest of the European cohort) in 27 European countries, by gender

	**Men (n=117 310)**		**Women (n=141 578)**	
	Crude model	Full adjusted model	Crude model	Full adjusted model
Austria	0·69 (0·56–0·86)	0·74 (0·60–0·91)	0·66 (0·58–0·75)	0·65 (0·57–0·74)
Bulgaria	1·00 (0·84–1·19)	0·93 (0·83–1·14)	1·03 (0·91–1·17)	1·06 (0·94–1·19)
Croatia	0·63 (0·50–0·79)	0·52 (0·42–0·65)	0·43 (0·36–0·53)	0·36 (0·30–0·44)
Cyprus	0·51 (0·39–0·66)	0·46 (0·36–0·58)	0·52 (0·43–0·64)	0·47 (0·39–0·56)
Czech Republic	0·34 (0·25–0·47)	0·26 (0·19–0·36)	0·43 (0·35–0·53)	0·35 (0·29–0·43)
Denmark	1·00 (0·83–1·20)	0·94 (0·79–1·13)	1·22 (1·08–1·38)	1·29 (1·14–1·45)
Estonia	0·97 (0·79–1·18)	0·63 (0·52–0·77)	1·07 (0·94–1·21)	0·75 (0·67–0·84)
Germany	1·85 (1·70–2·02)	1·88 (1·72–2·05)	1·54 (1·44–1·64)	1·74 (1·63–1·86)
Greece	0·52 (0·40–0·67)	0·44 (0·35–0·57)	0·53 (0·45–0·62)	0·41 (0·35–0·47)
Finland	0·99 (0·81–1·22)	0·89 (0·73–1·07)	0·72 (0·61–0·85)	0·77 (0·66–0·89)
France	0·97 (0·86–1·10)	0·98 (0·87–1·11)	1·21 (1·11–1·33)	1·20 (1·11–1·31)
Hungary	1·21 (1·03–1·42)	1·07 (0·93–1·24)	1·28 (1·14–1·43)	1·12 (1·01–1·24)
Iceland	1·36 (1·14–1·64)	1·19 (0·99–1·43)	1·81 (1·61–2·04)	1·42 (1·27–1·58)
Ireland	1·33 (1·13–1·57)	1·68 (1·38–1·88)	1·14 (1·00–1·30)	1·34 (1·19–1·52)
Italy	0·50 (0·44–0·57)	0·65 (0·57–0·73)	0·60 (0·55–0·65)	0·68 (0·63–0·74)
Latvia	0·61 (0·48–0·76)	0·46 (0·37–0·57)	0·76 (0·66–0·87)	0·55 (0·49–0·63)
Lithuania	0·36 (0·26–0·49)	0·35 (0·26–0·47)	0·52 (0·44–0·62)	0·45 (0·39–0·53)
Luxembourg	1·66 (1·40–1·97)	1·54 (1·28–1·84)	1·47 (1·29–1·67)	1·46 (1·28–1·66)
Malta	0·44 (0·32–0·61)	0·44 (0·32–0·61)	0·57 (0·46–0·70)	0·52 (0·42–0·64)
Norway	0·82 (0·68–0·98)	1·06 (0·89–1·26)	0·83 (0·71–0·96)	0·95 (0·83–1·10)
Poland	0·63 (0·55–0·72)	0·61 (0·54–0·69)	0·66 (0·61–0·72)	0·65 (0·59–0·71)
Portugal	0·93 (0·80–1·07)	0·87 (0·75–1·01)	1·74 (1·61–1·89)	1·40 (1·28–1·51)
Romania	0·77 (0·68–0·87)	1·13 (1·00–1·29)	0·62 (0·56–0·69)	0·85 (0·76–0·94)
Slovakia	0·37 (0·28–0·50)	0·26 (0·20–0·34)	0·41 (0·34–0·50)	0·31 (0·25–0·37)
Slovenia	0·78 (0·63–0·97)	0·78 (0·64–0·96)	0·92 (0·80–1·05)	0·90 (0·79–1·03)
Sweden	1·33 (1·15–1·54)	1·36 (1·19–1·56)	1·44 (1·29–1·62)	1·31 (1·18–1·47)
UK	1·29 (1·15–1·45)	1·16 (1·03–1·29)	1·12 (1·02–1·22)	1·07 (0·98–1·16)

Data are crude prevalence ratio or adjusted prevalence ratio, with 95% CI in parentheses. The adjusted prevalence ratio is adjusted for gender, age, country of birth, residence area, net monthly income of the household (equivalised for the different countries), long-standing illness, general activity limitation educational level, body-mass index, diet (fruits and vegetables consumption), smoking status, and days per week being physically active. All models were significant in relation to their respective null model (p<0·001).

## Discussion

To our knowledge, this study is one of the largest to specifically focus on comparing the prevalence of current depressive disorder by country in Europe that has included most countries and has used primary representative data of the general population in the communit**y**. We found that the overall prevalence of current depressive disorder in Europe is high (6·38%), with important variation across countries (ranging from 2·58% in the Czech Republic to 10·33% in Iceland). Prevalence was substantially higher among women than among men in all countries except Finland and Croatia. These results could be used as a baseline for further studies on the prevalence of current depressive disorder in Europe and to identify determinants for the observed cross-national variation.

Differences in the prevalence of depression have been observed in previous studies overall for the whole of Europe and by country.^4^, ^9^ Our estimates are lower than those of a previous meta-analysis by Lim and colleagues^9^ that reported an overall prevalence for the whole of Europe of 11·9%, but higher than WHO estimates of an overall prevalence of 4·2% for the European region.^4^ Notably, differences in the operational measure of depression between these previous reports and our study could be relevant to understand the variations found. Although we focused on current depressive disorder assessed via the PHQ-8, Lim and colleagues' meta-analysis used a wider definition of depression (ie, depressive symptoms using validated diagnostic or self-report instruments).^9^ Inclusion of individuals who do not meet the criteria for a diagnosis of major depression (eg, caused by bereavement, medical conditions, or substance use) or who have subclinical depression (eg, without functional impairment) might have led to an overestimation of prevalence in this meta-analysis. This potential overestimation was also suggested by the results of our sensitivity analysis using the SRD indicator, which provided a wider definition of both depression and temporal timeframe. By contrast, the WHO report^4^ used a narrower definition of depression based on International Classification of Diseases and DSM diagnostic codes. The loss of clinical cases through use of differing definitions is in general particularly problematic in prevalence studies, but the use of diagnostic codes specifically might lead to an underestimation of prevalence due to the possible exclusion of individuals with clinically relevant depression who have not received a diagnosis (eg, those without access to health services). Therefore, despite the potential cross-cultural differences between countries, because the operational measure of current depressive disorder used in our study is based on DSM criteria and also includes individuals with depression who might not have received a clinical diagnosis, our estimates could be considered to be a balanced and suitable estimation of the prevalence of current depressive disorder in Europe. These estimates could be used as a reference for the monitoring of the prevalence of depression in Europe. Additionally, they could provide a baseline for future epidemiological studies to determine at-risk groups that would support further research into determinants of health inequalities.

Our results show high variability in the prevalence of current depressive disorder between countries. Notably, based on the results of previous prevalence studies,^5^, ^6^, ^15^ some of the higher prevalences of depression were found in countries where we expected to find lower-than-average prevalences (eg, Iceland or Germany), and we found lower prevalences of depression in some countries where we expected to find higher-than-average prevalences (eg, Czech Republic or Lithuania). Although the summary estimates given for Europe hide between-country heterogeneity, both our geographically clustered analyses and previous research suggest that western European countries have a higher prevalence of depression.^9^, ^13^ Additionally, the small difference between crude and adjusted prevalence ratios in most of countries suggests that individual factors might have a small influence on the prevalence of current depressive disorder. Even though particular caution is needed in interpreting the results of geographically pooled clusters, both our findings and previous research suggest that demographic, cultural, and sociopolitical factors (eg, the variability between countries in population ageing, health-care access, work–life balance, job insecurity, and the increase of living costs) might be relevant determinants of the observed differences.^9^, ^13^, ^15^, ^23^, ^24^ These factors might have greater influence on the prevalence of current depressive disorders in countries with higher socioeconomic development (eg, Iceland or Germany) than in countries with moderate or lower socioeconomic development (eg, Czech Republic or Slovakia).^9^, ^23^ Therefore, the adoption of public health policies to try to minimise the effect of these factors on health (eg, through universal health coverage that adequately resources mental health care and public health preventive interventions) might reduce the prevalence of depression, especially in countries with higher prevalences of current depressive disorder and economic development.^10^, ^13^, ^23^ Nevertheless, further research including all European countries that considers both country-level and individual-level socioeconomic factors (including cultural and sociopolitical factors) could help us to better understand factors related to the prevalence of depression.

In terms of gender differences in current depressive disorder, clear differences in prevalence were found in all countries except Finland and Croatia. These results are in line with previous research,^11^, ^14^ particularly with the findings of a study based on European Social Survey (ESS) data by Huijts and colleagues.^15^ The results of Huijts and colleagues' study, which were stratified by gender, reported a higher overall prevalence of depressive symptoms using the Center for Epidemiological Studies-Depression scale (10·2% for men and 18·8% for women) than we report here, and they also showed that Finland had a smaller difference in prevalence between men and women than we identified here (6·7% men and 8·2% women).^15^ However, as was pointed out by Huijts and colleagues,^15^ although the prevalence was considered, cross-country comparisons were not done. Accordingly, because we identified countries with particularly high prevalences of current depressive disorder, our results might provide a reference for cross-country comparisons of the prevalence of current depressive disorder by gender in Europe, or a baseline for subsequent waves of EHIS and the ESS.

Our study had several limitations. First, data collection for EHIS-2 ended in 2015 and the contextual differences between countries in Europe could have since changed. Furthermore, different sampling methods were used to retrieve the information over a period of 3 years. Despite the delay between data collection and our analysis, our study is, to our knowledge, the most recent study based on primary data and covering the most EU countries. Therefore, our results could be a baseline for future comparisons at European-wide and country-wide levels using both subsequent waves of EHIS and other data. A further limitation relates to the use of the PHQ-8 to measure current depressive disorder rather than a clinical diagnostic interview, such as the Structured Clinical Interview for DSM or the The Composite International Diagnostic Interview. Additionally, different data collection methods were used across countries, although we had good comparability of the data and indicators from EHIS-2 between countries. We are aware that the PHQ-8 does not have perfect sensitivity and specificity to detect current depressive disorder,^19^, ^21^ but use of a cutoff score of 10 or higher on the PHQ-8 has previously been shown to have acceptable values when compared with these other instruments and also with other scoring methods (eg, the algorithm scoring method).^19^, ^20^, ^21^, ^25^ Moreover, the PHQ-8 is based on DSM criteria for depression and its balance of feasibility and accuracy makes it a useful general population indicator when using different data collection methods.^26^ Additionally, PHQ has been included in health surveys and medical records outside of Europe, potentially allowing worldwide comparisons, such as with the USA.^27^, ^28^ Nevertheless, given its metric properties, new studies to determine cutoff scores with higher sensitivity and specificity at population level could further increase the value of this tool. Another limitation is the high non-response rate between different countries, including some of those with the highest overall adjusted prevalence ratio.^16^ However, EHIS-2 used compensation methods for non-response rates to enable them to reach suitable ratios between the effective sample sizes and the minimum effective sample sizes.^16^ Therefore, although the estimates for these countries must be considered carefully, the potential non-response bias could be considered to be at least partially controlled. Additionally, individuals not living in private households are not captured in EHIS-2, which might have affected the representativeness of the study. Due to the higher rates of mental disorders among people not living in private households, such as those in prison or living in care homes, their exclusion could have led to an underestimation of the prevalence of current depressive disorder. However, because these populations comprise a small proportion of the European population, our results could be considered valid for most of the general population in the 27 included European countries. Additionally, the use of the Taylor linearisation method could lead to conservative estimations.^29^ Despite this potential limitation, because we aimed to compare prevalence between countries, and due to the potential implications that this comparison could have, we decided to go ahead with this method. Finally, some potentially relevant variables (eg, ethnicity or family history of depression) are not included in EHIS-2 and so we could not take them into account in our analyses. However, the covariates we did consider are usually included in all health surveys, allowing for comparison of results from different surveys.^13^, ^16^ Therefore, our analyses might be considered a valid and replicable approach to compare the prevalence of current depressive disorder between European countries.

In summary, we found that the overall prevalence of current depressive disorder in Europe is high, particularly among women, and that it varies widely between European countries, with an approximately four times difference in prevalence between the lowest-ranked and highest-ranked countries and with the greatest prevalences seen in countries with high economic development. These results could be used as a reference and baseline for the monitoring of the prevalence of current depressive disorder and highlight the need for the development of screening and preventive strategies for depression focused on the countries identified as having the highest prevalence and identified associated factors.

## Data sharing

Data from EHIS-2 are publicly available for research purposes under request to Eurostat.

## Declaration of interests

We declare no competing interests.

## References

[1] GBD Disease and Injury Incidence and Prevalence Collaborators (2018). Global, regional, and national incidence, prevalence, and years lived with disability for 354 diseases and injuries for 195 countries and territories, 1990–2017: a systematic analysis for the Global Burden of Disease Study 2017. Lancet.

[2] GBD 2017 DALYs and HALE Collaborators (2018). Global, regional, and national disability-adjusted life-years (DALYs) for 359 diseases and injuries and healthy life expectancy (HALE) for 195 countries and territories, 1990–2017: a systematic analysis for the Global Burden of Disease Study 2017. Lancet.

[3] Archer G, Kuh D, Hotopf M, Stafford M, Richards M (2020). Association between lifetime affective symptoms and premature mortality. JAMA Psychiatry.

[4] WHO (2017).

[5] Bromet E, Andrade LH, Hwang I (2011). Cross-national epidemiology of DSM-IV major depressive episode. BMC Med.

[6] Ferrari AJ, Somerville AJ, Baxter AJ (2013). Global variation in the prevalence and incidence of major depressive disorder: a systematic review of the epidemiological literature. Psychol Med.

[7] GBD 2016 Disease and Injury Incidence and Prevalence Collaborators (2017). Global, regional, and national incidence, prevalence, and years lived with disability for 328 diseases and injuries for 195 countries, 1990–2016: a systematic analysis for the Global Burden of Disease Study 2016. Lancet.

[8] Reibling N, Beckfield J, Huijts T, Schmidt-Catran A, Thomson KH, Wendt C (2017). Depressed during the depression: has the economic crisis affected mental health inequalities in Europe? Findings from the European Social Survey (2014) special module on the determinants of health. Eur J Public Health.

[9] Lim GY, Tam WW, Lu Y, Ho CS, Zhang MW, Ho RC (2018). Prevalence of depression in the community from 30 countries between 1994 and 2014. Sci Rep.

[10] Maske UE, Buttery AK, Beesdo-Baum K, Riedel-Heller S, Hapke U, Busch MA (2016). Prevalence and correlates of DSM-IV-TR major depressive disorder, self-reported diagnosed depression and current depressive symptoms among adults in Germany. J Affect Disord.

[11] Van de Velde S, Bracke P, Levecque K, Meuleman B (2010). Gender differences in depression in 25 European countries after eliminating measurement bias in the CES-D 8. Soc Sci Res.

[12] Arias de la Torre J, Vilagut G, Ronaldson A (2021). Prevalence and age patterns of depression in the United Kingdom. A population-based study. J Affect Disord.

[13] Rai D, Zitko P, Jones K, Lynch J, Araya R (2013). Country- and individual-level socioeconomic determinants of depression: multilevel cross-national comparison. Br J Psychiatry.

[14] Van de Velde S, Bracke P, Levecque K (2010). Gender differences in depression in 23 European countries. Cross-national variation in the gender gap in depression. Soc Sci Med.

[15] Huijts T, Stornes P, Eikemo TA, Bambra C (2017). Prevalence of physical and mental non-communicable diseases in Europe: findings from the European Social Survey (2014) special module on the social determinants of health. Eur J Public Health.

[16] Eurostat (2018). Quality report of the second wave of the European Health Interview survey: 2018 edition. EU. https://ec.europa.eu/eurostat/documents/7870049/8920155/KS-FT-18-003-EN-N.pdf/eb85522d-bd6d-460d-b830-4b2b49ac9b03.

[17] Eurostat (2013). European Health Interview Survey (EHIS wave 2): methodological manual: 2013 edition. EU. https://ec.europa.eu/eurostat/documents/3859598/5926729/KS-RA-13-018-EN.PDF/26c7ea80-01d8-420e-bdc6-e9d5f6578e7c.

[18] Kroenke K, Strine TW, Spitzer RL, Williams JBW, Berry JT, Mokdad AH (2009). The PHQ-8 as a measure of current depression in the general population. J Affect Disord.

[19] Moriarty AS, Gilbody S, McMillan D, Manea L (2015). Screening and case finding for major depressive disorder using the Patient Health Questionnaire (PHQ-9): a meta-analysis. Gen Hosp Psychiatry.

[20] Levis B, Benedetti A, Thombs BD (2019). Accuracy of Patient Health Questionnaire-9 (PHQ-9) for screening to detect major depression: individual participant data meta-analysis. BMJ.

[21] Wu Y, Levis B, Riehm KE (2020). Equivalency of the diagnostic accuracy of the PHQ-8 and PHQ-9: a systematic review and individual participant data meta-analysis. Psychol Med.

[22] Azur MJ, Stuart EA, Frangakis C, Leaf PJ (2011). Multiple imputation by chained equations: what is it and how does it work?. Int J Methods Psychiatr Res.

[23] Bambra C (2011). Health inequalities and welfare state regimes: theoretical insights on a public health ‘puzzle’. J Epidemiol Community Health.

[24] Buffel V, Van de Velde S, Bracke P (2015). The mental health consequences of the economic crisis in Europe among the employed, the unemployed, and the non-employed. Soc Sci Res.

[25] He C, Levis B, Riehm KE (2020). The accuracy of the Patient Health Questionnaire-9 algorithm for screening to detect major depression: an individual participant data meta-analysis. Psychother Psychosom.

[26] Pinto-Meza A, Serrano-Blanco A, Peñarrubia MT, Blanco E, Haro JM (2005). Assessing depression in primary care with the PHQ-9: can it be carried out over the telephone?. J Gen Intern Med.

[27] Tomitaka S, Kawasaki Y, Ide K (2018). Distributional patterns of item responses and total scores on the PHQ-9 in the general population: data from the National Health and Nutrition Examination Survey. BMC Psychiatry.

[28] Coley RY, Boggs JM, Beck A, Hartzler AL, Simon GE (2020). Defining success in measurement-based care for depression: a comparison of common metrics. Psychiatr Serv.

[29] West BT, McCabe SE (2012). Incorporating complex sample design effects when only final survey weights are available. Stata J.

