# Supplementary material for: Prevalence and variability of current depressive disorder in 27 European countries: a population-based study
Source: Lancet Public Health. 2021 May 4;6(10):e729–38. doi: 10.1016/S2468-2667(21)00047-5 (PMC8460452; doi:10.1016/S2468-2667(21)00047-5)
Supplement: Supplementary appendix [file mmc1.pdf]

THE LANCET
Public Health

Supplementary appendix

This appendix formed part of the original submission and has been peer reviewed. We post it as supplied by the authors.

Supplement to: Arias-de la Torre J, Vilagut G, Ronaldson A, et al. Prevalence and variability of current depressive disorder in 27 European countries: a population-based study. *Lancet Public Health* 2021; published online May 4. http://dx.doi.org/10.1016/S2468-2667(21)00047-5.

Contents:

Page 2. Supplementary file 1. Prevalence (%) of depression by country using different indicators.

Page 3. Supplementary file 2. Prevalence of positive (i.e. present more than half of days: score 2+) of Patient Health Questionnaire-8 (PHQ-8) items by country.

Page 4. Supplementary file 3. Prevalence ratios of current depressive disorder (PHQ-8≥10 country vs the rest).

Page 5. Supplementary file 4. Prevalence ratios (country vs the rest) calculated using the PHQ-8 algorithm scoring method and the self-reported indicator of depression.

Page 6 Supplementary file 5. Geographically clustered estimates of the prevalence of current depressive disorder and Prevalence Ratios (geographical cluster vs the rest)

Supplementary file 1. Prevalence (%) of depression by country using different indicators.

		PHQ-8 Algorithm		PHQ-8 Algorithm (major depression)		PHQ-8 Algorithm (other depression)		Self-reported depression		PHQ-8 Continuous score	
	**n**	**%**	**95% CI**	**%**	**95% CI**	**%**	**95% CI**	**%**	**95% CI**	**mean**	**95% CI**
**Overall**	258,888	7.01	6.86-7.16	2.98	2.88-3.08	4.03	3.92-4.15	6.94	6.80-7.08	2.77	2.74-2.79
**AT (Austria)**	15,701	5.23	4.65-5.81	1.44	1.13-1.75	3.79	3.29-4.29	7.41	6.77-8.05	2.67	2.60-2.74
**BG (Bulgaria)**	5,258	8.13	7.39-8.88	4.20	3.66-4.74	3.94	3.41-4.47	3.02	2.54-3.50	2.41	2.29-2.52
**CY (Cyprus)**	4,695	4.61	4.00-5.23	1.84	1.44-2.24	2.77	2.29-3.25	3.54	3.01-4.08	1.54	1.44-1.64
**CZ (Czechia)**	6,607	3.26	2.76-3.76	1.10	0.80-1.39	2.16	1.75-2.58	3.84	3.32-4.36	1.65	1.57-1.72
**DE (Germany)**	24,404	9.20	8.77-9.64	3.87	3.58-4.17	5.33	4.99-5.67	10.53	10.08-10.98	3.90	3.84-3.96
**DK (Denmark)**	5,449	6.67	5.95-7.38	3.19	2.68-3.69	3.48	2.95-4.00	7.81	7.06-8.57	3.09	2.98-3.20
**EE (Estonia)**	5,439	7.38	6.63-8.14	2.65	2.19-3.11	4.73	4.11-5.35	4.77	4.18-5.36	3.28	3.17-3.38
**EL (Greece)**	7,834	4.50	3.95-5.05	1.86	1.50-2.22	2.64	2.22-3.06	4.48	3.97-4.99	1.58	1.50-1.67
**FI (Finland)**	5,146	5.04	4.37-5.71	2.32	1.86-2.78	2.72	2.22-3.33	9.40	8.50-10.31	2.48	2.37-2.58
**FR (France)**	14,191	7.47	6.95-7.99	3.66	3.29-4.03	3.81	3.43-4.19	5.83	5.36-6.29	2.91	2.84-2.99
**HR (Croatia)**	5,016	3.91	3.36-4.46	1.52	1.18-1.86	2.39	1.96-2.83	5.59	4.95-6.22	1.63	1.55-1.72
**HU (Hungary)**	5,777	10.99	10.14-11.84	3.71	3.20-4.22	7.27	6.57-7.98	4.82	4.26-5.39	3.28	3.18-3.39
**IE (Ireland)**	9,046	6.21	5.48-6.95	3.19	2.69-3.69	3.02	2.47-3.58	11.15	10.29-12.01	3.08	2.96-3,19
**IS (Iceland)**	3,812	9.80	8.77-10.83	4.82	4.08-5.56	4.98	4.22-5.74	14.54	13.37-15.71	4.12	3.98-4.26
**IT (Italy)**	21,934	4.48	4.19-4.76	1.65	1.48-1.83	2.82	2.59-3.06	4.96	4.66-5.26	2.07	2.03-2.12
**LT (Lithuania)**	4,982	3.98	3.46-4.51	1.41	1.10-1.72	2.58	2.15-3.01	4.17	3.64-4.70	1.83	1.75-1.91
**LU (Luxembourg)**	3,629	8.95	7.96-9.93	4.79	4.05-5.52	4.16	3.47-4.85	9.02	8.07-9.97	3.97	3.83-4.11
**LV (Latvia)**	6,607	4.94	4.39-5.50	2.04	1.67-2.40	2.91	2.48-3.33	8.61	7.90-9.32	2.36	2.28-2.45
**MT (Malta)**	3,974	5.72	4.98-6.45	1.55	1.14-1.95	4.17	3.55-4.79	5.06	4.38-5.74	1.69	1.59-1.79
**NO (Norway)**	8,069	6.74	6.077.42	2.25	1.85-2.65	4.50	3.94-5.05	6.81	6.17-7.46	2.73	2.64-2.83
**PL (Poland)**	22,076	5.40	5.07-5.74	2.04	1.83-2.25	3.37	3.10-3.63	4.14	3.84-4.43	2.23	2.18-2.28
**PT (Portugal)**	17,974	10.63	9.96-11.29	3.93	3.52-4.34	6.69	6.16-7.23	11.77	11.09-12.45	3.23	3.13-3.32
**RO (Romania)**	16,422	4.72	4.36-5.07	2.07	1.84-2.30	2.64	2.37-2.92	1.47	1.26-1.68	2.31	2.25-2.37
**SE (Sweden)**	5,737	9.77	8.94-10.61	4.39	3.81-4.97	5.38	4.75-6.02	9.59	8.79-10.39	3.13	3.01-3.25
**SI (Slovenia)**	5,914	5.83	5.18-6.47	2.52	2.09-2.96	3.30	2.82-3.79	8.28	7.52-9.04	2.75	2.65-2.84
**SK (Slovakia)**	5,489	3.52	3.04-4.01	1.21	0.92-1.50	2.31	1.92-2.70	4.19	3.67-4.71	1.81	1.73-1.89
**UK (United Kingdom)**	17,706	8.59	8.04-9.13	4.00	3.61-4.39	4.59	4.18-4.99	9.05	8.51-9.60	2.57	2.49-2.65

n: number of individuals without weighting; PHQ-8: Patient Health Questionnaire 8 items; %: Prevalence (weighted); 95%CI: 95% Confidence Interval.
PHQ-Algorithm: either the first or second item (depressed mood or anhedonia) to be present "more than half the days" and at least 5 of the 8 symptoms to be present "more than half the days") or other depression (2 to 4 symptoms, including depressed mood or anhedonia, are required to be present "more than half the days").

Supplementary file 2. Prevalence of positive (i.e. present more than half of days: score 2+) of Patient Health Questionnaire-8 (PHQ-8) items by country

		Item 1		Item 2		Item 3		Item 4		Item 5		Item 6		Item 7		Item 8	
	**n**	**%**	**95%CI**	**%**	**95%CI**	**%**	**95%CI**	**%**	**95%CI**	**%**	**95%CI**	**%**	**95%CI**	**%**	**95%CI**	**%**	**95%CI**
**Total**	258,888	5.92	5.78-6.06	5.32	5.18-5.44	11.56	11.38-11.74	11.90	11.72-12.08	4.97	4.84-5.09	4.35	4.24-4.47	3.86	3.75-3.98	2.56	2.47-2.65
**AT**	15,701	5.24	4.73-5.76	3.48	3.00-3.96	12.81	12.08-13.54	10.44	9.77-11.12	3.88	3.43-4.34	1.98	1.64-2.32	2.58	2.21-2.95	1.68	1.38-1.99
**BG**	5,258	6.57	5.91-7.24	7.15	6.46-7.83	8.69	7.94-9.44	11.18	10.34-12.01	3.82	3.31-4.33	5.79	5.15-6.42	4.64	4.08-5.21	4.92	4.35-5.50
**CY**	4,695	3.77	3.22-4.32	4.47	3.87-5.08	5.41	4.75-6.07	5.43	4.76-6.09	2.06	1.63-2.49	2.34	1.88-2.81	1.84	1.46-2.23	1.25	0.94-1.57
**CZ**	6,607	3.67	3.13-4.21	2.42	1.98-2.86	6.84	6.20-7.48	7.31	6.59-8.02	2.11	1.72-2.50	1.96	1.57-2.35	2.03	1.64-2.42	0.96	0.70-1.22
**DE**	24,404	7.61	7.22-8.00	5.92	5.57-6.26	18.20	17.64-18.75	15.82	15.29-16.35	7.00	6.64-7.37	5.58	5.25-5.91	5.23	4.91-5.56	3.13	2.87-3.39
**DK**	5,449	5.53	4.88-6.17	4.74	4.15-5.34	11.65	10.77-12.53	11.67	10.76-12.58	5.11	4.48-5.74	6.14	5.46-6.82	4.58	3.98-5.17	1.94	1.55-2.33
**EE**	5,439	6.04	5.38-6.71	5.76	5.10-6.41	14.86	13.87-15.84	16.00	14.98-17.02	5.91	5.25-6.57	2.86	2.39-3.33	3.56	3.05-4.07	2.65	2.21-3.08
**EL**	7,834	3.60	3.12-4.08	4.12	3.61-4.63	5.85	5.26-6.45	6.08	5.48-6.69	2.47	2.05-2.90	2.43	2.03-2.82	2.59	2.18-3.01	2.24	1.85-2.62
**FI**	5,146	4.46	3.85-5.07	3.74	3.17-4.31	8.38	7.58-9.19	8.68	7.86-9.51	3.51	2.97-4.04	5.78	5.08-6.47	2.59	2.11-3.06	1.28	0.96-1.60
**FR**	14,191	6.01	5.55-6.48	5.61	5.16-6.06	12.93	12.29-13.56	12.73	12.11-13.35	6.63	6.15-7.10	5.65	5.21-6.09	4.36	3.96-4.77	2.42	2.13-2.71
**HR**	5,016	2.94	2.47-3.40	3.59	3.06-4.12	6.56	5.87-7.25	7.40	6.66-8.14	1.99	1.60-2.37	1.93	1.52-2.33	1.96	1.57-2.35	1.98	1.62-2.35
**HU**	5,777	8.80	8.05-9.54	8.92	8.17-9.66	17.16	16.16-18.16	18.83	17.79-19.87	4.47	3.92-5.02	5.75	5.14-2.82	3.41	2.94-3.88	2.84	2.40-3.28
**IE**	9,046	3.85	3.31-4.38	5.90	5.22-6.57	12.39	11.44-13.34	13.20	12.25-14.16	6.99	6.26-7.72	5.90	5.24-6.47	4.69	4.11-5.26	2.80	2.30-3.30
**IS**	3,812	8.06	7.16-8.96	6.79	5.96-7.61	17.07	15.85-18.30	21.82	20.48-23.17	9.17	8.21-10.12	6.82	5.99-7.65	8.14	7.25-9.04	5.14	4.41-5.86
**IT**	21,934	3.68	3.42-3.93	3.59	3.33-3.84	8.63	8.25-9.02	8.19	7.82-8.57	2.92	2.69-3.15	2.26	2.05-2.47	2.48	2.27-2.69	1.69	1.51-1.86
**LT**	4,982	2.61	2.19-3.03	4.28	3.74-4.82	7.14	6.46-7.82	6.98	6.30-7.66	1.77	1.41-2.13	1.79	1.43-2.15	1.51	1.20-1.83	1.00	0.74-1.25
**LU**	3,629	7.41	6.53-8.29	6.27	5.47-7.08	16.80	15.55-18.04	16.07	14.86-17.29	7.83	6.93-8.73	7.56	6.67-8.44	6.12	5.32-6.93	3.37	2.78-3.97
**LV**	6,607	4.48	3.96-4.99	3.65	3.18-4.12	11.76	10.95-12.57	9.95	9.19-10.71	2.56	2.15-2.96	4.11	3.60-4.62	2.51	2.12-2.90	2.75	2.34-3.16
**MT**	3,974	12.24	11.19-13.28	4.14	3.51-4.77	4.85	4.17-5.53	4.28	3.63-4.93	2.74	2.20-3.27	2.26	1.78-2.74	1.77	1.33-2.20	1.74	1.31-2.18
**NO**	8,069	8.70	7.99-9.42	4.02	3.50-4.55	10.56	9.77-11.35	11.05	10.24-11.85	5.49	4.91-6.08	2.93	2.49-3.37	3.66	3.17-4.14	3.09	2.66-3.53
**PL**	22,076	3.94	3.65-4.22	4.86	4.55-5.18	8.41	8.00-8.82	8.77	8.35-9.20	2.36	2.13-2.60	3.21	2.94-3.47	2.46	2.22-2.69	2.26	2.05-2.48
**PT**	17,974	8.97	8.36-9.58	8.82	8.23-9.42	16.56	15.76-17.35	13.95	13.22-14.69	4.57	4.13-5.02	5.49	5.00-5.98	4.51	4.07-4.95	3.19	2.81-3.57
**RO**	16,422	3.68	3.37-3.99	4.30	3.96-4.64	4.90	4.54-5.26	5.80	5.41-6.19	2.65	2.39-2.92	2.25	2.01-2.50	2.34	2.09-2.58	2.15	1.91-2.38
**SE**	5,737	9.29	8.50-10.08	7.19	6.49-7.90	13.90	12.96-14.83	15.17	14.20-16.13	7.43	6.72-8.14	6.62	5.95-7.30	5.63	5.00-6.26	3.13	2.66-3.60
**SI**	5,914	5.20	4.60-5.80	4.12	3.58-4.65	12.38	11.50-13.27	11.93	11.05-12.81	3.98	3.46-4.50	4.34	3.80-4.88	3.51	2.99-4.02	2.75	2.3-3.19
**SK**	5,489	3.10	2.65-3.55	3.37	2.90-3.84	7.05	6.40-7.70	7.68	6.98-8.38	1.76	1.42-2.11	1.67	1.33-2.01	1.60	1.27-1.93	1.62	1.30-1.95
**UK**	17,706	7.71	7.20-8.22	6.53	6.06-7.00	8.42	7.80-8.96	14.66	13.98-15.34	6.69	6.21-7.17	5.39	4.96-5.83	5.13	4.70-5.55	3.41	3.06-3.75

n: number of individuals without weighting; %: Prevalence (weighted); 95%CI: 95% Confidence Interval; AT: Austria; BG: Bulgaria; CY: Cyprus; CZ: Czechia; DE: Germany; DK: Denmark; EE: Estonia; EL: Greece; FI: Finland; FR: France; HR: Croatia; HU: Hungary; IE: Ireland; IS: Iceland; IT: Italy; LT: Lithuania; LU: Luxembourg; LV: Latvia; MT: Malta; NO: Norway; PL: Poland; PT: Portugal; RO: Romania; SE: Sweden; SI: Slovenia; SK: Slovakia; UK: United Kingdom.

Supplementary file 3. Prevalence ratios of current depressive disorder (PHQ-8≥10 country vs the rest).

	Crude model		Full adjusted model	
	PR	95% CI	aPR	95% CI
**AT (Austria)**	0.67	0.60-0.75	0.68	0.61-0.76
**BG (Bulgaria)**	1.02	0.92-1.14	1.02	0.93-1.12
**CY (Cyprus)**	0.52	0.44-0.61	0.46	0.40-0.53
**CZ (Czechia)**	0.40	0.34-0.47	0.32	0.27-0.38
**DE (Germany)**	1.64	1.56-1.73	1.80	1.71-1.89
**DK (Denmark)**	1.13	1.02-1.25	1.15	1.05-1.27
**EE (Estonia)**	1.04	0.94-1.16	0.70	0.56-0.78
**EL (Greece)**	0.52	0.46-0.60	0.42	0.37-0.48
**FI (Finland)**	0.82	0.72-0.93	0.82	0.72-0.92
**FR (France)**	1.12	1.04-1.20	1.12	1.04-1.20
**HR (Croatia)**	0.51	0.43-0.59	0.41	0.36-0.48
**HU (Hungary)**	1.26	1.15-1.38	1.09	1.00-1.19
**IE (Ireland)**	1.20	1.09-1.34	1.44	1.31-1.58
**IS (Iceland)**	1.62	1.47-1.79	1.33	1.21-1.47
**IT (Italy)**	0.56	0.52-0.60	0.67	0.62-0.72
**LT (Lithuania)**	0.47	0.40-0.55	0.41	0.36-0.48
**LU (Luxembourg)**	1.53	1.38-1.69	1.50	1.35-1.66
**LV (Latvia)**	0.72	0.64-0.81	0.52	0.46-0.58
**MT (Malta)**	0.51	0.43-0.61	0.49	0.41-0.58
**NO (Norway)**	0.81	0.73-0.91	0.99	0.89-1.11
**PL (Poland)**	0.66	0.61-0.71	0.64	0.59-0.68
**PT (Portugal)**	1.45	1.35-1.56	1.21	1.13-1.30
**RO (Romania)**	0.68	0.62-0.73	0.94	0.87-1.02
**SE (Sweden)**	1.38	1.26-1.51	1.34	1.26-1.46
**SI (Slovenia)**	0.86	0.77-0.97	0.85	0.76-0.95
**SK (Slovakia)**	0.40	0.34-0.47	0.28	0.24-0.33
**UK (United Kingdom)**	1.19	1.11-1.28	1.10	1.03-1.18

PR: Prevalence Ratio. aPR: Prevalence Ratio adjusted for gender, age, country of birth, residence area, net monthly income of the household (equivalised for the different countries), long standing illness, general activity limitation educational level, body mass index, diet (fruits and vegetables consumption), smoking status and days per week doing sport; 95%CI: 95% Confidence Interval; All models were significant in relation to their respective null model ($p<0.001$).

Supplementary file 4. Prevalence ratios (country vs the rest) calculated using the PHQ-8 algorithm scoring method and the self-reported indicator of depression.

	PHQ-8 algorithm				Self-reported depression			
	Crude model		Full adjusted model		Crude model		Full adjusted model	
	PR	95% CI	aPR	95% CI	PR	95% CI	aPR	95% CI
**AT (Austria)**	0.74	0.66-0.83	0.78	0.70-0.88	1.07	0.98-1.17	1.02	0.94-1.11
**BG (Bulgaria)**	1.16	1.06-1.28	1.13	1.03-1.23	0.43	0.37-0.51	0.46	0.39-0.54
**CY (Cyprus)**	0.66	0.57-0.65	0.55	0.49-0.62	0.51	0.44-0.59	0.47	0.40-0.54
**CZ (Czechia)**	0.45	0.39-0.53	0.37	0.32-0.43	0.55	0.48-0.63	0.45	0.39-0.51
**DE (Germany)**	1.42	1.35-1.50	1.64	1.55-1.73	1.74	1.66-1.83	1.78	1.69-1.87
**DK (Denmark)**	0.95	0.85-1.06	1.02	0.92-1.13	1.12	1.02-1.24	1.12	1.02-1.24
**EE (Estonia)**	1.05	0.95-1.17	0.73	0.66-0.80	0.69	0.61-0.78	0.48	0.43-0.55
**EL (Greece)**	0.64	0.56-0.72	0.48	0.43-0.54	0.64	0.56-0.72	0.55	0.50-0.62
**FI (Finland)**	0.72	0.63-0.82	0.74	0.65-0.84	1.36	1.23-1.50	1.27	1.16-1.40
**FR (France)**	1.08	1.00-1.16	1.07	1.00-1.14	0.82	0.75-0.89	0.84	0.77-0.91
**HR (Croatia)**	0.56	0.48-0.64	0.45	0.39-0.52	0.80	0.72-0.90	0.70	0.62-0.78
**HU (Hungary)**	1.59	1.47-1.72	1.42	1.32-1.53	0.69	0.61-0.78	0.64	0.570.71
**IE (Ireland)**	0.89	0.79-1.00	1.09	0.97-1.21	1.61	1.49-1.75	1.95	1.81-2.11
**IS (Iceland)**	1.40	1.26-1.56	1.25	1.13-1.38	2.10	1.93-2.28	1.83	1.68-1.99
**IT (Italy)**	0.60	0.56-0.65	0.69	0.64-0.73	0.68	0.64-0.73	0.87	0.82-0.93
**LT (Lithuania)**	0.57	0.50-0.65	0.49	0.43-0.53	0.60	0.53-0.68	0.54	0.48-0.62
**LU (Luxembourg)**	1.28	1.14-1.43	1.29	1.15-1.44	1.30	1.17-1.45	1.24	1.12-1.38
**LV (Latvia)**	0.70	0.63-0.79	0.50	0.45-0.56	1.24	1.14-1.35	0.94	0.86-1.02
**MT (Malta)**	0.82	0.72-0.93	0.71	0.62-0.82	0.72	0.64-0.83	0.70	0.61-0.80
**NO (Norway)**	0.96	0.89-1.06	1.23	1.11-1.35	0.98	0.89-1.09	1.14	1.04-1.25
**PL (Poland)**	0.75	0.71-0.81	0.71	0.67-0.76	0.57	0.53-0.62	0.55	0.51-0.59
**PT (Portugal)**	1.54	1.44-1.64	1.23	1.15-1.32	1.73	1.62-1.84	1.50	1.41-1.60
**RO (Romania)**	0.66	0.61-0.71	0.86	0.79-0.92	0.20	0.18-0.36	0.30	0.26-0.45
**SE (Sweden)**	1.41	1.29-1.54	1.41	1.29-1.53	1.39	1.27-1.52	1.34	1.23-1.46
**SI (Slovenia)**	0.83	0.74-0.93	0.84	0.75-0.93	1.19	1.09-1.31	1.18	1.08-1.30
**SK (Slovakia)**	0.50	0.43-0.57	0.36	0.32-0.42	0.60	0.52-0.68	0.45	0.40-0.51
**UK (United Kingdom)**	1.27	1.18-1.36	1.19	1.121.27	1.37	1.28-1.46	1.21	1.14-1.29

PR: Prevalence Ratio. aPR: Prevalence Ratio adjusted for gender, age, country of birth, residence area, net monthly income of the household (equivalised for the different countries), long standing illness, general activity limitation educational level, body mass index, diet (fruits and vegetables consumption), smoking status and days per week doing sport; 95%CI: 95% Confidence Interval; All models were significant in relation to their respective null model ($p<0.001$).

Supplementary file 5. Geographically clustered estimates of the prevalence of current depressive disorder and Prevalence Ratios (geographical cluster vs the rest)

	Prevalence		Crude model		Full adjusted model	
	%	95% CI	PR	95% CI	aPR	95% CI
**North**	6.45	6.15-6.76	1.01	0.96-1.07	0.97	0.92-1.02
**South**	4.48	4.27-4.70	0.66	0.62-0.69	0.67	0.64-0.71
**East**	4.54	4.37-4.71	0.66	0.63-0.69	0.61	0.59-0.64
**West**	7.92	7.67-8.18	1.65	1.58-1.72	1.73	1.66-1.81

North: Denmark, Estonia, Finland, Iceland, Lithuania, Latvia, Norway, Sweden; South: Cyprus, Greece, Italy, Malta, Portugal; East: Bulgaria, Czechia, Croatia, Hungary, Poland, Romania, Slovakia, Slovenia; West: Austria, France, Germany, Ireland, Luxembourg, United Kingdom; PR: Prevalence Ratio. aPR: Prevalence Ratio adjusted for gender, age, country of birth, residence area, net monthly income of the household (equivalised for the different countries), long standing illness, general activity limitation educational level, body mass index, diet (fruits and vegetables consumption), smoking status and days per week doing sport; 95%CI: 95% Confidence Interval; All models
